# Radiographic Evaluation of a Modular Anterior Lumbar Interbody Fusion (ALIF) Cage: Subsidence and Segmental Lordosis

**DOI:** 10.7759/cureus.109522

**Published:** 2026-05-23

**Authors:** Harvinder Bhatti, Nathan R Wanderman, Jessica L Shellock, Richard D Guyer, Donna D Ohnmeiss, Kelly Van Schouwen, Nicholas Spina

**Affiliations:** 1 Orthopedic Surgery, Atlanta Spine &amp; Orthopaedics, Atlanta, USA; 2 Spine Surgery, Twin Cities Orthopedics, Woodbury, USA; 3 Spine Surgery, Center for Disc Replacement, Texas Back Institute, Plano, USA; 4 Research, Texas Back Institute Research Foundation, Plano, USA; 5 Research, Research Source, Austin, USA; 6 Orthopedics, University of Utah Health, Salt Lake City, USA

**Keywords:** alif, anterior lumbar interbody fusion, lordosis, lumbar spine, modular cage, spine surgery, subsidence

## Abstract

Introduction: Anterior lumbar interbody fusion (ALIF) is an established intervention for degenerative pathology and sagittal plane corrections. Despite advances in implant technology, challenges such as vertebral endplate damage, implant subsidence, and loss of segmental lordosis remain. A modular ALIF cage, designed with an endplate-first insertion technique, aims to protect endplates, reduce insertional trauma, and enable precise restoration of disc height and lordosis. The purpose of this study was to evaluate early radiographic outcomes after implantation of a modular ALIF device, specifically examining the incidence of implant subsidence and changes in segmental lordosis.

Methods: A multicenter retrospective analysis of 23 patients was conducted to evaluate the Axis-ALIF Modular Cage (Axis Spine Technologies Ltd, Leeds, UK) implanted in a total of 27 levels. ALIF was undertaken primarily for the treatment of symptomatic disc degeneration unresponsive to nonoperative care. Radiographs were obtained preoperatively, at early postoperative follow-up, and at final follow-up (range: 3 to 24 months; mean: 6.6 months). These images were evaluated by an independent core lab using an automated image analysis platform to assess cage subsidence and segmental lordosis.

Results: No radiographic evidence of subsidence was observed in the 24 levels with complete paired imaging data, with the mean change in disc height being 0.002 mm (range: -0.69 to 0.78 mm). Segmental lordosis increased by approximately 8° immediately postoperatively and remained stable at follow-up (mean change: +0.02°).

Conclusions: In this small retrospective cohort, the modular ALIF implant demonstrated consistent early radiographic findings, with no observed subsidence and maintained segmental lordosis. These findings support further prospective evaluation in larger controlled studies.

## Introduction

Anterior lumbar interbody fusion (ALIF) is a well-established fusion option. The anterior approach to the lumbar spine provides broad access to the disc space, allowing placement of single large, lordotic implants. Advancements in ALIF technology continually evolve through improved graft materials and fusion cage designs. While there have been significant advancements, issues such as damage to the vertebral body endplates, implant subsidence, and the potential for loss of lordosis remain known challenges associated with ALIF cages. To improve osseointegration, recent research has focused on enhancing the surface characteristics of fusion cages, including techniques such as surface texturing and coating. Despite these efforts, studies have shown that impacting fusion cages into the disc space can produce problems such as abrasion, delamination, or the production of wear debris [1-4].

Damage to the vertebral endplates may play a role in cage subsidence, which in turn may impact sagittal alignment, a key consideration since ALIF is frequently performed to improve sagittal balance. Subsidence may be particularly important in patients experiencing symptoms caused by disc height loss due to degeneration with related foraminal narrowing and stenosis. ALIF has demonstrated effectiveness in providing significant improvement in symptoms through indirect decompression [5-7]. For the benefit of indirect decompression to be sustained, the interbody cage must maintain the restored disc height and not undergo significant subsidence. A systematic review found that the median rate of patients with post-ALIF subsidence was 12.8%, with the lowest reported rate being 6% and the highest 23.1% [8].

As discussed by Leveque et al., sagittal alignment has long been a focus in spinal deformity surgery but has often been overlooked in short-segment fusion for painful degenerative conditions of the lumbar spine [9]. In a series of more than 500 lumbar fusion patients, the authors found that over 25% were malaligned both preoperatively and postoperatively. A systematic review found that ALIF can be effective in correcting spinal malalignment [7].

One of the goals of ALIF is to restore the height of a degenerated, collapsed disc space. With traditional monobloc fusion cages, high impact and/or aggressive preparation of the disc space is often needed to position the cage between the vertebral bodies. These maneuvers may gouge the endplates with the cage or instruments, initiating a cascade of endplate injury, weakening, and device subsidence. A recent innovation in ALIF cage design aimed at reducing vertebral body endplate damage involves the use of a modular cage system. The modular cage is implanted with just the device endplates first being positioned into the prepared disc space without high impaction needed. After the device endplates are positioned in the disc space, the core is inserted between them to gain height and the desired angulation without further contacting or manipulating the vertebral body endplates. This study was conducted to evaluate the radiographic outcomes of a modular ALIF cage, with a specific focus on implant subsidence rates and changes in segmental lordosis.

## Materials and methods

Implant design

**Figure 1:**
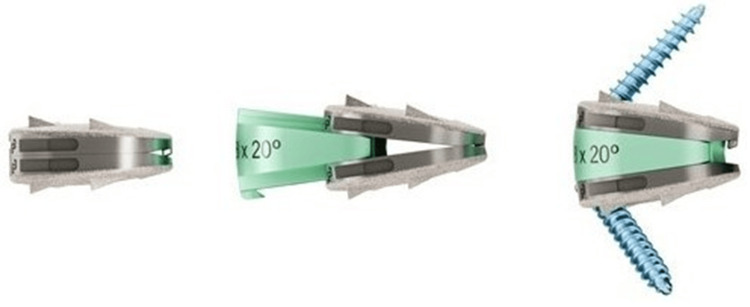
Modular ALIF cage The modular device consists of titanium alloy endplates with textured surfaces and a titanium alloy core packed with bone graft, which is inserted between the endplates after they have been positioned within the disc space. The cage is secured with screws inserted into the superior and inferior vertebral bodies. This figure was provided by and reproduced with permission from Axis Spine Technologies (Leeds, UK) [10]. ALIF: Anterior lumbar interbody fusion.

The cage evaluated in this study was the Axis-ALIF Modular Cage (Axis Spine Technologies Ltd, Leeds, UK), which is designed for intraoperative adaptability to enhance placement positioning, correction accuracy, endplate protection, and implant-to-bone coverage of the vertebral endplates. This modular ALIF cage implant employs an “endplate-first” approach, where the titanium alloy endplates are positioned into the disc. Then, a core featuring a chamber for bone graft material is inserted between the endplates to achieve the desired implant height and angulation (Figure 1). The implants come in posterior heights of 6, 8, and 10 mm and lordotic angles of 10^o^, 15^o^, 20^o^, 25^o^, 30^o^, 35°, and 40°. The implant design allows for a low-profile insertion of the primary implant, which helps protect the vertebral endplates. Bone graft material is loaded into the implant core before it is positioned between the endplates, with the option to add more graft material afterward if needed.

Surgical technique considerations

All procedures were performed using a standard anterior retroperitoneal approach to the lumbar spine. Disc preparation was performed using conventional techniques with emphasis on the removal of disc material while preserving the subchondral bony endplates. The modular implant was inserted using an endplate-first approach, in which low-profile endplates were positioned within the disc space before insertion of the central core. The core was then introduced to achieve the desired posterior height and segmental lordosis. This approach is designed to facilitate controlled insertion within a contained working corridor and to reduce the risk of excessive endplate violation during implantation.

Patient population

This retrospective study included 23 adult patients who underwent one- or two-level ALIF using a modular ALIF implant system. ALIF was undertaken primarily for the treatment of symptomatic disc degeneration unresponsive to nonoperative care. Five experienced spine surgeons from four centers across the United States performed surgeries on a total of 27 spinal levels.

Details of the surgical procedures and availability of each imaging data point are detailed in Table 1. At the L5-S1 level, in 13 of 15 cases (86.7%), a core of 15° or greater lordosis was used.

**Table 1:** Overview of the surgeries performed and radiographs available for measurement

Patient	Levels	Footprint (width x depth, mm)	Device posterior height (mm)	Angle (^o^)	Posterior fixation	Subsidence measured	Lordosis measured
1	L3-L4	38 x 28	6	10	Yes	Yes	Yes
	L4-L5	38 x 28	8	10	Yes	Yes	Yes
2	L4-5	38 x 28	8	10	Yes	Yes	Yes
3	L4-5	38 x 28	10	10	Yes	Yes	Yes
4	L4-5	38 x 28	6	10	Yes	No	No
5	L5-S1	38 x 28	8	20	Yes	Yes	Yes
6	L5-S1	38 x 28	10	15	Yes	Yes	Yes
7	L4-5	38 x 28	10	10	Yes	Yes	Yes
	L5-S1	38 x 28	8	20	Yes	Yes	Yes
8	L5-S1	38 x 28	8	20	Yes - single side only	Yes	Yes
9	L5-S1	38 x 28	8	15	No	Yes	Yes
10	L4-5	38 x 28	10	10	Yes	Yes	Yes
	L5-S1	38 x 28	8	20	Yes	Yes	Yes
11	L5-S1	38 x 28	8	20	No	Yes	Yes
12	L5-S1	41 x 31	8	15	Yes	Yes	Yes
13	L5-S1	38 x 28	6	10	No	Yes	Yes
14	L4-5	38 x 28	10	10	Yes	Yes	Yes
	L5-S1	38 x 28	6	15	Yes	Yes	Yes
15	L4-5	38 x 28	6	10	No	No	No
16	L5-S1	41 x 31	10	15	No	No	Yes
17	L5-S1	38 x 28	6	10	Yes	Yes	Yes
18	L5-S1	38 x 28	6	20	Yes	Yes	No
19	L5-S1	38 x 28	8	15	Yes	Yes	No
20	L5-S1	38 x 28	8	20	Yes	Yes	No
21	L5-S1	38 x 28	8	15	Yes	Yes	No
22	L5-S1	38 x 28	6	20	Yes	Yes	Yes
23	L5-S1	38 x 28	6	20	Yes	Yes	Yes

Radiographic assessment

Standing anteroposterior and lateral lumbar spine radiographs were obtained preoperatively, early postoperatively, and at the most recent follow-up (between 3 and 24 months).

All radiographic measurements were conducted at an independent imaging core lab using FXA software (functional X-ray analysis, RAYLYTIC Software GmbH, Leipzig, Germany), an AI-assisted image analysis platform previously validated for the assessment of spinopelvic parameters and disc height measurements on radiographic imaging. The FXA system performs automated image registration using grayscale optimization algorithms, allowing precise alignment of serial radiographs and enabling quantification of changes in radiographic parameters over time.

The measurement precision of the FXA method for absolute disc height has been reported to be approximately 0.04 mm, corresponding to an estimated error of ~0.06 mm for calculated changes between time points. Furthermore, FXA-derived measurements have demonstrated excellent agreement (intraclass correlation coefficient (ICC) preoperative range: 0.85-0.92, postoperative range: 0.81-0.87) with human-generated measurements in the assessment of spinopelvic parameters on radiographic imaging. These values are derived from prior validation studies [11].

Disc height was measured at each operated level from neutral lateral radiographs and recorded as the average of the anterior, middle, and posterior disc space heights. Segmental lordosis was recorded by measuring the Cobb angle of the operated segment(s). There were 27 operated levels, and disc height data are reported for 24 levels. For two patients, the long-term follow-up radiographs were not available for mean disc height measurement. For one patient, the disc height on the immediate postoperative image was not readable.

Definitions

In this study, subsidence was defined as a reduction of at least 1 mm in disc height between the early postoperative and final postoperative radiographs. Given the known limitations of radiographic imaging resolution, this threshold was selected to exceed the estimated measurement error of the FXA system and to align with commonly used definitions in the literature [12]. The initial change in segmental angulation was defined as the change in segmental lordosis from the preoperative radiograph to the early postoperative radiograph. The preservation of lordosis was evaluated by comparing segmental lordosis values from the early postoperative measurement to those obtained at the final follow-up.

## Results

Subsidence

No subsidence, defined as a decrease in disc height of 1 mm or greater, was observed in any of the 24 operated levels with both early and final follow-up radiographs. The mean disc height values are provided in Table 2. The mean change in disc height between early and final follow-up was 0.002 mm (range: -0.69 to 0.78 mm).

**Table 2:** Disc height measurements at early postoperative and final follow-up evaluations There was no indication of subsidence based on changes in disc space height between early postoperative and final follow-up radiographic measurements (p > 0.99), and no level showed a change in height of >1 mm. All measurements are reported in mm.

Variable	Early postop	Final follow-up
Number of levels	24	24
Mean	14.02	14.03
Standard deviation	2.17	2.3
Median	14.07	14.18
Minimum	8.49	7.83
Maximum	18.18	17.9

Segmental lordosis

Prior to surgery, the mean segmental lordosis of the level undergoing fusion was 18.8°. At the early postoperative visit, the segmental lordosis was significantly increased to 26.8° (p < 0.001). This alignment was maintained at final follow-up, with a mean increase of 0.02° between early and final follow-up.

Complications and re-operations

No intraoperative complications related to implant insertion were reported. No revision surgeries were documented during the available follow-up period.

Case example

**Figure 2:**
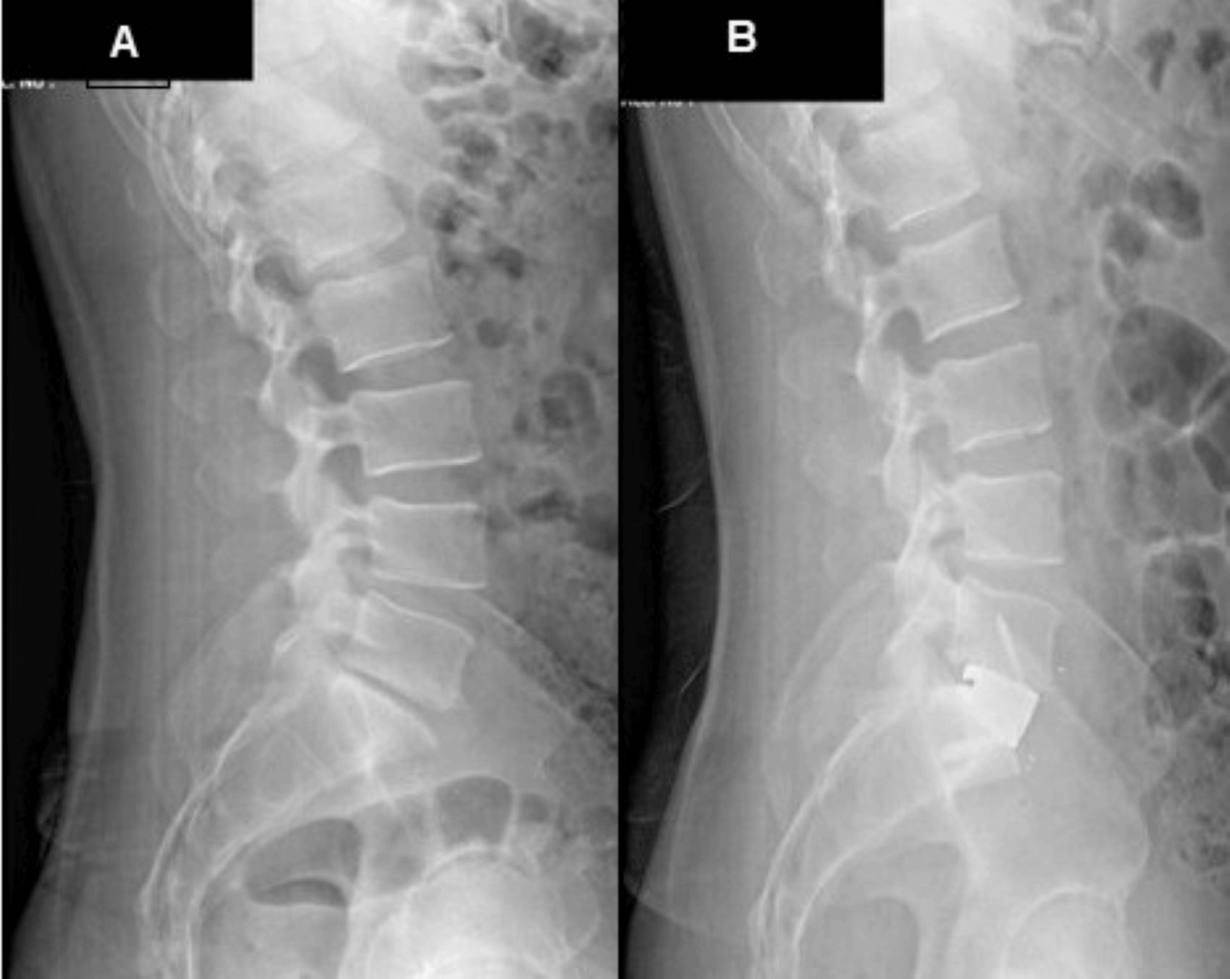
Preoperative and postoperative radiographs after ALIF using the modular cage Preoperative (A) and postoperative (B) radiographic images of a patient with a modular cage at L5-S1. The disc space height increased from 5.3 mm preoperatively to 13.9 mm at 4 days postoperatively using an 8 mm core and measured 13.5 mm at 6-month follow-up. With respect to segmental lordosis, the preoperative Cobb angle was 14.8°, which increased to 26.9° at the early postoperative visit following insertion of the 20° core. The angle measured at the six-month follow-up was 26.0°. ALIF: Anterior lumbar interbody fusion.

Figure 2 provides an example of a patient who underwent ALIF with the modular cage at L5-S1. The disc space height increased from 5.3 mm preoperatively to 13.9 mm at four days post-surgery using an 8 mm height core. The disc space height at six-month follow-up was 13.5 mm. With respect to segmental lordosis, preoperatively, the Cobb angle was 14.8°, which increased to 26.9° at the early postoperative visit after insertion of the 20° core. The angulation at six-month follow-up was 26.0°.

## Discussion

This study demonstrated that use of a modular, endplate-first ALIF cage is associated with consistent early radiographic findings, including absence of observed subsidence and maintenance of increased segmental lordosis in a small retrospective cohort.

In a biomechanical investigation, a unique study design was used in which a modular cage was compared to the same device implanted as a static cage to compare the amount of endplate damage done during device implantation [13]. The modular device produced significantly less endplate gouging than the cage in static form. It was also found that damage to the endplates was related to an increased risk of anterior cage migration. Using a similar format of comparing the modular cage to a static version of the same cage, a study using cadaveric specimens and a more refined methodology also assessed endplate damage during device implantation [14]. While some endplate damage occurred with both implant types, more severe damage penetrating the endplate and damaging the bone was more common with the static implant. The authors noted that this type of endplate damage and associated subsidence were related to reduced gains in lordosis.

In this current study, the threshold for classifying an ALIF level was considered to have subsidence if there was a decrease in radiographic disc space height of more than 1 mm between early follow-up and final follow-up, a stricter standard than what is typically applied in most other research studies [12]. Even with these more rigorous criteria, none of the operated levels exhibited subsidence. Preventing or reducing subsidence could help preserve the disc space and maintain foraminal height, minimizing the risk of symptom recurrence by maintaining indirect decompression.

Subsidence has been associated with an increased rate of subsequent adjacent segment surgery following ALIF [15]. Anterior placement of the cage within the disc space has been identified as an independent risk factor for subsidence after ALIF, regardless of patient demographic factors [15]. The modular cage design is intended to reduce the risk of endplate damage during insertion, especially in discs that have experienced significant collapse at the posterior aspect.

The current study found that the modular ALIF cage created and maintained segmental lordosis at the operated level(s). The importance of sagittal alignment in patients undergoing lumbar fusion for painful degenerative conditions has recently become more appreciated [9,16,17]. Several studies have found that inadequate restoration of lordosis was associated with adjacent segment degeneration [17-19]. Two of these studies reported that this resulted in an increased rate of re-operation for this condition. The other study reported that they did not identify a definitive link between lordosis and patient-reported clinical outcomes.

The use of hyperlordotic cages (those with an angulation greater than 15°) at the lower lumbar levels for ALIF to increase lordosis and improve sagittal alignment has gained acceptance [16]. However, inserting these highly angled implants can be difficult, as the disc space is often small and narrowed. Achieving adequate posterior placement of the cage to enhance posterior disc height can be challenging. Impaction of a hyperlordotic cage in a narrowed space may also exert greater force on the endplates, increasing the risk of endplate damage, which can lead to subsidence and subsequent reduction in segmental lordosis [20-22]. Another potential problem associated with the use of hyperlordotic cages is that, due to trigonometric constraints, the fixed anterior height results in a relatively short posterior height, particularly at steeper lordotic angles. This may contribute to iatrogenic foraminal stenosis, which has been associated with the onset of new L5 radiculopathy in approximately 17% of patients [23]. Symptom onset was found to be significantly associated with greater increases in lordosis and less posterior disc height after fusion. Modular ALIF cages may offer a potential approach to mitigate this issue by allowing an endplate-first insertion followed by the introduction of a core that independently sets the desired posterior cage height, irrespective of lordotic angle. This modular approach helps maintain foraminal height, thereby reducing the risk of iatrogenic foraminal stenosis compared with static hyperlordotic cages.

Key limitations of this pilot study include the small sample size, lack of a control group, incomplete demographic and bone health data, and reliance on radiographic measurements rather than CT-based assessment. As a retrospective pilot study, this analysis was limited by the availability of consistent data. Of 27 treated levels, 24 had complete paired early and final radiographic data to include in the primary subsidence analysis. Three levels were excluded due to incomplete imaging follow-up. One of the strengths of the study was the use of precise measurement techniques for disc space height and segmental lordosis. The cases included contributions from five spine surgeons from four different centers. The study results were consistent, with all patients exhibiting less than 1 mm of subsidence and no loss of lordosis throughout the follow-up period. More definitive evaluation of the modular cage would come from prospective studies using CT-based evaluation of subsidence and fusion, as well as clinical outcome and randomized comparison to other ALIF cages, which would require a substantially larger patient population.

## Conclusions

These findings demonstrate the feasibility of the modular ALIF approach and consistent early radiographic outcomes in a small cohort, with all patients exhibiting less than 1 mm of subsidence and no loss of lordosis throughout the follow-up period. Further prospective, controlled studies are required to determine comparative clinical performance relative to conventional ALIF cage designs.
